# NK Cell-Based Immunotherapy and Therapeutic Perspective in Gliomas

**DOI:** 10.3389/fonc.2021.751183

**Published:** 2021-10-26

**Authors:** Changqing Pan, You Zhai, Guanzhang Li, Tao Jiang, Wei Zhang

**Affiliations:** ^1^ Department of Neurosurgery, Beijing Tiantan Hospital, Capital Medical University, Beijing, China; ^2^ Beijing Neurosurgical Institute, Capital Medical University, Beijing, China; ^3^ China National Clinical Research Center for Neurological Diseases, Beijing, China; ^4^ Chinese Glioma Genome Atlas (CGGA) and Asian Glioma Genome Atlas (AGGA), Beijing, China

**Keywords:** natural killer cells, alloreactivity, chimeric antigen receptor, adoptive cell immunotherapy, glioma

## Abstract

Glioma is the most common malignant primary brain tumor diagnosed in adults. Current therapies are unable to improve its clinical prognosis, imposing the need for innovative therapeutic approaches. The main reason for the poor prognosis is the great cell heterogeneity of the tumor and its immunosuppressive microenvironment. Development of new therapies that avoid this immune evasion could improve the response to the current treatments. Natural killer (NK) cells are an intriguing candidate for the next wave of therapies because of several unique features that they possess. For example, NK cell-based immunotherapy causes minimal graft-*versus*-host disease. Cytokine release syndrome is less likely to occur during chimeric antigen receptor (CAR)-NK therapy, and CAR-NK cells can kill targets in a CAR-independent manner. However, NK cell-based therapy in treating glioma faces several difficulties. For example, CAR molecules are not sufficiently well designed so that they will thoroughly release functioning NK cells. Compared to hematological malignancies, the application of many potential NK cell-based therapies in glioma lags far behind. Here, we review several issues of NK cells and propose several strategies that will improve the efficacy of NK cell-based cancer immunotherapy in the treatment of glioma.

## Introduction

Gliomas are the most common intracranial primary malignant tumor (1). The incidence of gliomas is approximately of six cases per 100,000 individuals worldwide. Glioblastoma (GBM), the most common glioma histology, has a 5-year relative survival of ∼5%. While the majority of cases are sporadic, a small portion of these tumors are associated with neurofibromatosis type I, tuberous sclerosis, and Li-Fraumeni syndrome. Standard medical care, including the most extensive tumor resection followed by radiotherapy and chemotherapy. Surgery is commonly performed with both diagnostic and therapeutic intent. The therapeutic goal of surgery is to remove as much tumor tissue while preserving neurological function. Even for diffuse gliomas, a biopsy is recommended to acquire tissue specimens for molecular profiling (IDH mutations,1p/19q codeletion, MGMT promoter methylation, EGFR amplification et al) (2). Most patients receive chemotherapy. Classic schemes including Stupp (NCT00006353) and PCV (Procarbazine, CCNU, and Vincristine) (3). The strategies of radiotherapy are determined by the disease subtype and prognostic factors, including residual tumor volume, age, KPS. The details of novel strategies including tumor-treating fields (TTFields), checkpoint inhibitor, vaccine and oncolytic virus are described in **Table 1**. In general, the prognosis of high-grade glioma is still unpleasant, which calls for more efficient approaches.

**Table 1:** Applications of novel strategies in glioma.

Strategies	Interventions	Results	Tumors	Phase	Reference/NCT
TTFields	TTFields plus temozolomide VS temozolomide alone	TTFields plus temozolomide improve PFS and OS significantly	Glioblastoma	III	NCT00916409
Checkpoint Inhibitor	nivolumab VS bevacizumab	Nivolumab failed to improve OS	Recurrent Glioblastoma	III	NCT02017717
	Neoadjuvant pembrolizumab	Neoadjuvant extended OS and enhanced both the local and systemic antitumor immune response	Recurrent Glioblastoma	/	Cloughesy et al. (4)
Vaccine	Rindopepimut (CDX-110), a vaccine targeting EGFRvIII	Rindopepimut did not increase OS	Glioblastoma	III	NCT01480479
	peptide vaccine (IDH1-vac) targeting mutant IDH1	IDH1-vac increased PFS and immune responses, but accompanied by a high frequency of pseudoprogression	Grade III and IV Astrocytomas	I	NCT02454634
	autologous dendritic cell vaccine ICT-107	ICT-107 significantly improved PFS	Glioblastoma	II	NCT01280552
Oncolytic Virus	intratumoral infusion of polio-rhinovirus chimera (PVSRIPO)	PVSRIPO therapy increased OS	Recurrent malignant Glioma	I	NCT01491893
	intratumoral injection of oncolytic adenovirus (DNX-2401)	DNX-2401 resulted in dramatic responses with long-term survival	Recurrent malignant Glioma	I	NCT00805376

Adoptive cell therapy (ACT), especially CAR-armed cell therapy, has great potential due to its high cytotoxicity and precise strikes. ACT consists of a series of infusions of autologous or allogeneic immune cells to kill targets, and T cell-based immunotherapy is an example of a mainstream form of ACT that is well-studied. Chimeric antigen receptor (CAR) T cells targeting CD19 is one therapy that has resulted in encouraging success in patients with B cell malignancies and has been approved by the US Food and Drug Administration (FDA) (5–7). However, there are numerous logistic and clinical limitations to the use of autologous CAR-modified T cells. Personalized CAR-T products are time-consuming and expensive to produce. Allogeneic T cell-based therapy can cause substantial toxic effects, such as graft-*versus*-host disease (GvHD) and cytokine release syndrome (CRS) (8). Furthermore, the results of CAR-T cell therapy for solid tumors are suboptimal. These shortcomings of CAR-T cells have called for interest in other candidate.

NK cells are a subpopulation of the innate immune system (9, 10). NK cells can be identified by CD3(-) CD56(+). Depending on the level of CD56 and CD16 expression, NK cells can be divided into CD16^+^CD56^dim^ and CD16^−^CD56^bright^ cells. CD16^+^CD56^dim^ NK cells predominate in peripheral blood while CD16^−^CD56^bright^ NK cells are distributed into secondary lymphoid organs (11). CD16^−^CD56^bright^ NK cells are robust cytokine producers and are weakly cytotoxic while the CD16^+^CD56^dim^ NK cell population can mediate serial killing of infected and/or malignant cells. NK cell receptors are germline-encoded without a requirement for ‘V(D)J’ recombination. Natural killer (NK) cells have gained attention as a promising alternative candidate for ACT owing to their unique biological attributes.

NK cells do not require any prior antigen and can rapidly recognize and kill cells for which major histocompatibility complex (MHC) class I molecular expression is compromised by infection or transformation (9). Once activated, NK cells can release perforin and granzyme, contributing to target cell lysis. NK cells upregulate death ligands on their surface, such as FAS ligand and TRAIL, and initiate the caspase pathway of tumor cells and induce apoptosis when binding to death receptors on target cells. NK cells can eradicate cancer cells through antibody-dependent cellular cytotoxicity (ADCC) mediated by FcγRIIIA/CD16a. Furthermore, NK cells produce interferon gamma (IFN-γ), regulating and activating the adaptive immune response.

NK cell-based therapy is safe and has potential generated as off-the-shelf cellular therapy products. Autologous NK cells exert limited cytotoxicity against autologous tumors, while allogeneic NK cells are highly cytotoxic and cause minimal risk of GvHD (12–16). Thus, NK cells can originate from different sources, such as peripheral blood NK cells (PBNK), induced pluripotent stem cells (iPSCs), umbilical cord blood (UCB) and NK-92 cells, and this eliminates the need to produce a personalized CAR-NK product. However, the claim that allogeneic NK cells cause no or minimal GvHD and CRS is controversial and originated from observations obtained during clinical trials, especially in the setting of hematopoietic cell transplantation (HCT), the mechanism of which has not been thoroughly discussed. Here, we review important issues regarding NK cells and glioma and discuss several options that can be used to improve the efficacy of CAR-NK in glioma treatment.

## The Safety of NK Cell-Based Immunotherapy

### NK Cell Alloreactivity

All NK cells are non-responsive towards healthy autologous cells, which involves the interaction of at least inhibitory killer immunoglobulin-like receptors (KIRs) or CD94-NKG2A with one autologous MHC class I molecule. KIRs can be classified based on two factors: the number of immunoglobulin-like domains (2D and 3D) and the length of the intracytoplasmic tail (L or S). Inhibitory KIRs usually possess a long cytoplasmic tail (KIR2DL), whereas activating KIR possess a short one (KIR2DS), except for the activating KIR2DL4, which has a long cytoplasmic tail. Inhibitory KIRs contain immunoreceptor tyrosine-based inhibition motif (ITIM) sequences responsible for the inhibitory signal. Unlike cytotoxic CD8+ T cells, which are highly specific for antigens, NK cells express clonally distributed inhibitory receptors termed KIRs that recognize determinants (KIR ligands) shared by subsets of HLA-B or -C allotypes (17–20). More than fifty KIR family members have been identified, and each of these genes is highly polymorphic and has thousands of alleles (21). Three subfamilies and associated inhibitory specificities are well determined (**Table 2**). The CD94-NKG2A heterodimer, belonging to C-type lectins, is specific for HLA-E (24–26).

**Table 2:** Three subfamilies of KIRs and specific ligands.

Group^a^	HLA-class I specificity^b^
KIR2DL1(CD158a)	C2(-Cw2, -Cw4, -Cw5, -Cw6)
KIR2DL2/3(CD158b1/b2)	C1(-Cw1, -Cw3, -Cw7, -Cw8)
KIR3DL1(CD158e1)	Bw4(-B27, -B51)

a Each group compromises different numbers of alleles, which differ by 1-9 nucleotide substitutions (22).

b Two groups of HLA-C alleles are distinguished by dimorphic positions Ser 77–Asn 80 (C1) and Asn 77–Lys 80 (C2) of the α1 helix (23). HLA-B allotypes share the Bw4 sequence motif at positions 77–83 of the α1 helix (22).

**Figure 1:**
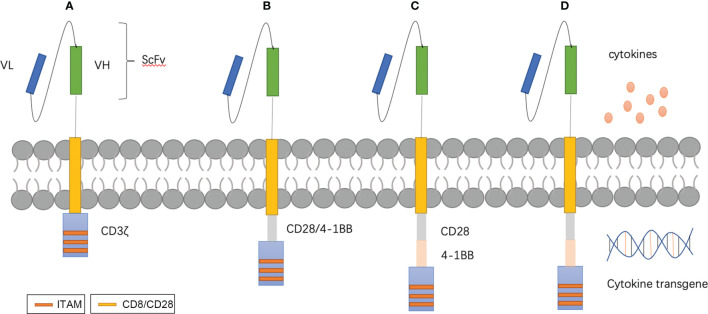
T-CAR designs. **(A–D)** show the four generations of T-CAR. In brief, CAR contains three parts: extracellular domains are comprised of a single-chain variable fragment (scFv) for recognizing targeted antigen and transmembrane domains, and endocellular domains for transducing signals. First generation CARs consist of the basic structure with CD3ζ **(A)**. Second generation CARs contain an additional costimulatory domain such as CD28 or 4–1BB **(B)**. Third generation CARs possess multiple costimulatory domains **(C)**. Fourth-generation CARs, also known as ‘armored CARs’ can be designed to secret cytokines to improve the proliferation, persistence.

Major models used to predict NK cell alloreactivity include ‘missing self’ and ‘missing ligand’ (**Figure 1**). Missing self-recognition (the ‘ligand–ligand’ model) was proposed by Karre et al. and occurs under HLA haplotype-mismatched transplants in the graft-*versus*-host direction (27). Donor NK cells express a KIR for the self HLA class I group that is absent in the recipient, which mediates alloreactions (28–30). HLA testing is required to predict NK cell alloreactivity due to the missing self-model.

Because the genes for KIR, HLA, and CD94–NKG2 are located on different chromosomes (31–33), KIR genes segregate independently of the HLA genes, and thus, KIR mismatches can exist in two HLA-matched individuals. Also, it was found that many individuals have 3 inhibitory KIRs (for HLA-C1 and -C2 and for HLA-Bw4 alleles), while their own cells only express 1 or 2 HLA KIR ligands (22, 34, 35). KIR expression is donor specific, but not related to the donor or recipient HLA and is not affected by the recipient’s HLA groups (36). The missing ligand model (the ‘receptor-ligand’ model) was based on these. According to this model, NK cell alloreactivity occurs not only in HLA haplotype-mismatched transplants, but also in HLA haplotype-matched transplants from donors possessing ‘extra’ KIR(s), for which neither donor nor recipient possess HLA ligand(s) (35–37). The donor’s potentially self-reactive NK cells can trigger an alloreactive effect in the recipient while maintaining anergy in the donor. Analysis of the KIR expression on the donor’s NK cells and HLA testing of the recipient’s cells are required to predict NK cell alloreactivity due to the missing ligand model.

The missing self and missing ligand models can be used to predict NK cell alloreactivity. Although alloreactive NK cells can eradicate tumor cells, the anti-tumor effect is not confined to alloreactive NK cells. NK cell activity depends on the balance between inhibitory and stimulatory receptors. An anti-tumor effect can be mediated by NK cells expressing stimulatory receptors, such as activating KIR and NKG2D (38–40).

## NK Cell-Based Immunotherapy Causes Minimal GvHD and CRS

GvHD refers to a condition resulting from the systemic attack of allogenic T cells on recipient tissues after allogeneic hematopoietic stem cell transplantation or infusion of allogeneic T cells (41–43). The effects of GvHD are commonly manifested in the gastrointestinal tract, liver, and skin (44), and severe GvHD can be fatal. The role of alloreactive NK cells on GvHD in the setting of HCT varies among studies. Some investigations found that alloreactive NK cells were related to decreased GvHD (36, 45, 46), which was partially attributed to the observations that allogeneic NK cells would be expected to kill host dendritic cells (DCs) and donor T cells (46, 47). Miller et al. analyzed 2,062 patients undergoing unrelated donor HCT (48). They found that one or more KIR ligands were missing *versus* the presence of all ligands, which is associated with a low relapse rate in patients with early myeloid leukemia. This omission predicted a greater risk of developing grade 3-4 GvHD in the setting of chronic myeloid leukemia (CML) patients. Miller et al. attributed the higher rate of acute GvHD in CML to the expanded myeloid pool with more host antigen-presenting cells (APCs) capable of presenting alloantigen to donor T cells (48).

HCT after ablation of bone marrow is used to cure hematological malignancies and results in less cancer relapse compared to chemoradiotherapy (49). T cells of allogeneic hematopoietic grafts for treating leukemia mediate the antileukemia effect as well as lethal GvHD. In many studies, it was attempted to prevent GvHD by depleting the T cells from the graft and infusing large numbers of hematopoietic stem cells to overcome rejection (50), which was at the expense of immunity reconstitution failure and infection. Later, NK cells from alloreactive donors were found to protect patients against rejection and GvHD in the setting of HCT (46). Interestingly, we found the idea that NK cell-based therapy caused GvHD mostly happened in the setting of HCT. But we should not evaluate the effects of alloreactive NK cells on GvHD in the setting of HCT because the effect of T cells in the grafts is negligible. It is likely that T cell interference is the most important controversial element with respect to the alloreactive NK cell effects on GvHD.

In fact, NK cell-based immunotherapy is safe and causes minimal GvHD. GvHD most likely occurs when NK cells from donors with several KIR subfamilies are infused into recipients possessing one group HLA ligand. Valiante et al. analyzed NK cell receptor repertoires in the peripheral blood of two human donors (donor PP only possessed group 1 HLA-C ligand, and donor NV possessed group 1 and 2 HLA-C ligands and the Bw4 HLA-B ligand, both of which have three KIR subfamilies as demonstrated in **Table 2**) (51). They found that more than 98% of NK clones were inhibited self-HLA class I allotypes, and no NK cell from either donor was able to lyse the autologous B cell line (51). Interestingly, NV possessed approximately 15% of the analyzed NK cell clones, did not express KIR2DL2 or CD94:NKG2a, and was able to lyse the B cell line from PP, whereas the NK cell clones from PP failed to lyse the B cell line from NV (51). Ruggeri adopted functional analysis to evaluate the NK cell alloreactivity in more than 200 NK clones (46). Alloreactivity was defined as positive when the frequency of lytic clones was no less than 1 in 50 (46). In addition, the expression of CD94:NKG2a is inversely related to KIR levels (51). Approximately, 50% of NK cells in an individual express CD94:NKG2a (51, 52). Cell-surface HLA-E expression depends on many peptides, including the leader peptides of HLA-A, -B, or -C, and downregulation of HLA-E expression requires the elimination of three types of HLA molecules (53, 54). Thus, NK cells expressing CD94–NKG2A display no alloreactivity because all individuals express HLA-E molecules. Therefore, NK cell-based immunotherapy is safe most of the time and will cause minimal GvHD because alloreactive NK cells only account for a small proportion. In addition, healthy cells express high levels of MHC class I molecules, but they express no or minimal level of ligands for NK cell activating receptors. Conversely, tumorigenic cells downregulate MHC class I expression but upregulate the expression of ligands for NK cell activating receptors. For example, MICA/MICB and ULBP, ligands for NKG2D, are often induced by stress or transformation (55, 56). The integration of the activating and inhibitory signals from the ligand/receptor determines NK cell activity. Some studies indicated that the positive signal delivered by NKG2D could override inhibition. Therefore, NK cells become alloreactive prior to killing tumor cells.

CRS involves elevated levels of circulating cytokines, especially interferons and immune-cell hyperactivation, which manifests as an influenza-like syndrome, organ failure, and even death (57). CAR-NK is less likely to induce CRS and neurotoxicity partially because of a different spectrum of secreted cytokines consisting of activated NK cells that produce IFN-gamma and GM-CSF, and CAR-T cells that predominantly release tumor necrosis factor (TNF)-a and interleukins, such as IL-1, IL-2, and IL-6 (57, 58). The mechanism was validated by clinical trials. Liu et al. launched a clinical trial (NCT03056339) that administered HLA-mismatched anti-CD19 CAR-NK cells to 11 patients with high-risk lymphoid malignancies (16). The administration of CAR-NK cells was not associated with the development of cytokine release syndrome and there was no increase in the levels of inflammatory cytokines, including interleukin-6, over baseline (16).

## NK Expansion Techniques

Large numbers of cells are essential for successful adoptive transfer cell therapy. It has been proved that high doses of NK cells from10^7cells/kg to 4.7×10^10 total NK cells can be well tolerated (16, 59, 60). NK cells only account for approximately 10% of peripheral blood mononuclear cells. The NK92 cell line is used in current clinical trials with CAR-NK because of their unlimited proliferation ability *in vitro*. However, the NK92 cell line is tumorigenic and lacks CD16 and NKp44 expression, and additionally, these cells will lose their proliferation ability due to lethal irradiation infusion (61, 62). Because of these drawbacks, it is unlikely that they will be an ideal cell source for CAR-NK cell therapy.

Expansion protocols possess considerable heterogeneity. The expansion process often takes 2-3 weeks of culture in the presence of mitogenic cytokines, and engineered feeder cells can optimize the expansion process. K562 cells engineered to express membrane-bound IL-15 or IL-21 along with the adhesion molecule 4-1BBL are adopted as feeder cells in many clinical trials (63, 64). There have been reports of 300-fold expansions combined with IL-2 and IL-15. IL-15 is important to NK cell survival and function (65, 66), and 4-1BBL provides a cell to cell contact-dependent co-stimulatory signal (67). We observed that low density less than 10^5cells/mL is not ideal for NK expansion. We recommend that the ratio of engineered K562 feeder cells and NK cells is 1:1 to 2:1. The K562 cell line possesses unique properties and lacks HLA expression. As described above, inhibitory KIRs recognizing corresponding HLA ligands can inactivate NK cells. This can be proved by our laboratory findings that other cell lines engineered to express IL-21 and 4-1BBL failed to achieve high-fold NK expansion. Some studies used RetroNectin-stimulated T (RN-T) cells as feeder cells (68, 69). Briefly, the procedure for this is to culture T cells from autologous peripheral blood mononuclear cells (PBMCs) with RetroNectin and anti-CD3 monoclonal antibody. In approximately 2 weeks, RN-T cells can serve as feeder cells after irradiation. RetroNectin plays a role in cell adhesion (70). The anti-CD3 monoclonal antibody leads to T-cell activation and cytokine secretion, and activated T cells express ligands of NKG2D (47, 71). Irradiated PBMCs as feeder cells were utilized by Parkhurst et al. and share a similar mechanism with RN-T in NK cell expansion (60, 72). The CD14+ monocyte fraction of irradiated PBMCs function in cell-cell contact (73). Feeder cells derived from autologous PBMCs eliminate the need for infusion of viable malignant feeder cells into the NK cell product. Feeder-free expansion approaches have also been tested. Li et al. cultured NK cells in an anti-CD16 (Beckman Coulter)-coated flask (74). Antibody-coated beads targeting CD2 and NKp46 (CD335) are commercially available. However, not everyone or each NK cell expresses these stimulatory receptors, and feeder-free expansion protocols lose cell-to-cell contact effects. For example, only a part of NK cells of an individual expresses CD16 on blood NK cells (75, 76).

## Adoptive Cell Therapy and CAR-NK Cells

There are several developmental stages in ACT. Lymphokine-activated killer (LAK) cells, which consist of a mixture of NK cells, NKT cells, and T cells, were adopted to overcome insufficient quantities of immune cells (77). An unwanted side effect of a high dose of IL-2 is that it induces capillary leak syndrome and neuropsychiatric diseases in a manner similar to that of CRS. To obtain immune cells that can effectively respond to tumors, Rosenberg introduced the concept of tumor-infiltrating lymphocytes (TILs) (78), which have many similarities with LAK cells except the origin of lymphocytes. The former is isolated from the stroma of tumors, while the latter is acquired from PBMCs. Success with TILs has been achieved in many solid tumors, including breast cancer tumors (79, 80). With TCR engineering, tumor-specific TCR α and β chains are identified and integrated with T cells *via* viral vectors (81). There must be specificity with T-cell engineering and CAR-T. Compared to CAR-T therapy, there are limited choices for physiological receptors with TCR engineering. Effective CAR-T cell therapy relies on optimal CAR molecular design. The CAR construct is becoming increasingly sophisticated with the understanding of T cell activation and tumor-specific and -associated antigens (82). Four generations of CAR designs have been developed that are mainly different in categories and number of co-stimulation factors (**Figure 1**). For safety and effectiveness, many novel designs have been tested, such as ‘inverted CAR,’ ‘off-switch CAR’ and ‘logic-gate CAR’ (83–85).

**Figure 2:**
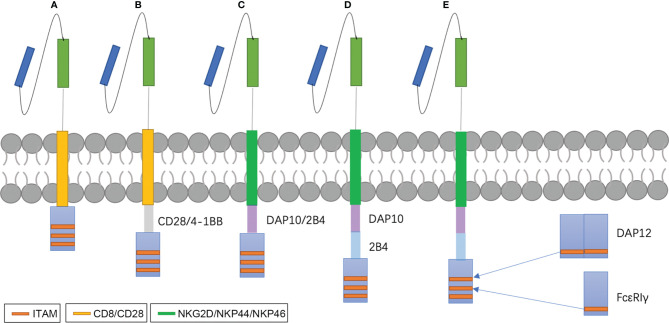
NK-CAR designs. Early studies exploring CAR-NK cells used CAR constructs optimized for T cell signaling and function **(A, B)**. Later, many investigators begin to study costimulatory domains specific for NK cell signaling, such as DAP10, DAP12 or 2B4 **(C, D)**. DAP10 is the adaptor molecule of NKG2D and acts *via* a Syk-independent regulatory pathway (92). DAP12 is the adaptor molecules of NKG2C, NKp44 and activating killer immunoglobulin receptors (KIRs) and contain immunoreceptor tyrosine-based activation motifs (ITAMs) (93). 2B4, an important co-stimulation factor of NK activation, contains immunoreceptor tyrosine-based switch motifs (ITSMs) that recruit adaptor molecules such as SLAM-associated protein (SAP) to mediate signal transduction (94). We design a new CARs that replace ITAMs of CD3ζ with FcϵRIγ and DAP12 **(E)**.

NK cell-based immunotherapy is a subset of ACT and is similar to adoptive T therapy, especially with respect to CAR therapy. CAR-NK-related clinical trials show that the most adopted CAR design corresponds with first and second generation T-CAR (86). Most NK-CARs use CD28 and 4-1BB, which are more specific to T cells, as their transmembrane and intracellular domain, respectively (87, 88). Later studies began to design CARs specific to NK cells. For example, intracellular domains replaced CD28 with 2B4, DAP12, or DAP10 (89, 90). Li et al. proved that the signaling domains of CAR-NK, such as NKG2D-2B4, exhibited superior *in vitro* and *in vivo* anti-tumor activities compared to that which contains CD28-4-1BB (91). The revolution of CAR-NK therapy is described in **Figures 2A–D**, and it is clear that the CAR construct is becoming increasingly sophisticated with the growing understanding of T cell activation and tumor-specific and -associated antigens that are also suitable for NK cells.

## Immunosuppressive Mechanisms of Glioma

Glioma, especially GBM, shows an extreme cell heterogeneity, diffuse growth patterns and high invasiveness. Gliomas were thought to be “immune cold” tumors with low infiltration of lymphocytes (95). Furthermore, microenvironment of the glioma has the ability to suppress immune response systematically and locally.

Mahaley et al. first reported the presence of lymphopenia in GBM (96). Studies have found patient-derived peripheral blood lymphocytes shows immune defects that exhibited varying levels of proliferative unresponsiveness to the T-cell mitogens concanavalin A (ConA), phytohemagglutinin (PHA) and anti-CD3 monoclonal antibody as well as with the T-dependent B-cell mitogen, pokeweed mitogen (PWM) (97, 98). A selective impairment of the IL-2 system and T cell receptor-mediated signaling in lymphocytes of patients with glioblastomas may contribute to the unresponsiveness (99, 100). Compared to healthy individuals, accumulation of myeloid-derived suppressor cells (MDSCs) in the peripheral blood of patients with glioma was found (101, 102). MDSCs impair tumor immunity by interacting with macrophages to increase IL-10 and decrease IL-12 production, driving a tumor-promoting type 2 response (103). Inhibitory soluble factors secreted by glioma can suppress lymphocyte’s function. Transforming growth factor beta (TGF-β) can impair peripheral blood NK cell function by downregulating NKG2D (104–107). Therefore, the immune responses are suppressed systematically in glioma microenvironment.

Gliomas often overexpress phosphorylated signal transducer and activator of transcription 3 (p-STAT3) that induces a variety of immunosuppressive factors including IL-10, prostaglandin E2 (PGE2), vascular endothelial growth factor (VEGF) and TGF-β (108). These soluble factors can suppress cytotoxic T lymphocytes activity and proliferation (108). TGF-β and IL-10 can induce Tregs that inversely modulate immune response (109). Chemokines and cytokines, such as CX3CL1 and CCL5 can recruit tumor-associated macrophages to GBM microenvironment and contribute to abnormal angiogenesis (110, 111). Most GBM cells express high levels of MHC class I molecules that can inhibit NK cells by interacting with inhibitory KIRs (112). Absolute survival advantages of tumor depriving of nutrition and oxygen might suppress NK cell metabolism and antitumor activity (113). N6-methyladenosine (m6A) modification is an emerging field in the study of tumorigenicity and therapy resistance of glioma (114, 115). The relationship between m6A states and immune infiltration and function in glioma is still unclear. Studies found higher m6Ascore was associated with T cells exhaustion and lower NK cells in the m6Ascore-high pancreatic ductal adenocarcinoma (116). So, the immune responses are suppressed locally in glioma microenvironment.

## Applications of NK Cells For Glioma Treatment

Except for unique advantages of NK cells, there is still a potential of NK cells in treating glioma. Cózar et al. analyzed RNA-seq datasets from the TCGA database and found NK-cell infiltration in both low grade glioma and GBM and even had higher scores compared to T-cell infiltration, which paved the way for the use of treatments targeting NK cells in glioma (117). Similar to adoptive T therapy, NK cell-based immunotherapy mainly concentrates on hematological malignancies. Thus far, the therapeutic utility of NK cell-based immunotherapy for the treatment of glioma has mainly been investigated in preclinical studies (**Table 3**). These trials most utilize either PBNK cells or NK92 cells, as well as first and second generation CARs designed for T cells and not optimized for NK cell signaling. Furthermore, clinical trials pay more attention to evaluate the safety of CAR-NK therapy. Although preclinical studies began to test the efficiency of DAP12 specific for NK cell signaling. Most of them adopted similar CARs as used in clinical trials. Compared to hematological malignancies, both in the quantity and CARs design, the application of NK cell-based therapies in glioma lags far behind.

**Table 3:** Applications of NK cells in glioma.

Targets	NK Source	CAR construct	Tumors	Phase	Reference/NCT
EGFRvIII	NK92 cell line	scFv-CD28TM+IC-CD3ζ	Glioblastoma	**/**	Genßler et al. (118)
EGFRvIII	YTS NK cell line	scFv-DAP12 TM+IC	Glioblastoma	**/**	Müller et al. (90)
EGFRvIII	KHYG-1 NK cell line	scFv-CD28 TM+IC-CD137-CD3ζ	Glioblastoma	**/**	Murakami et al. (119)
EGFRvIII	NK92 cell line	scFv-CD28 TM+IC-CD3ζ	Glioblastoma	**/**	Han et al. (120)
HER2	NK92 cell line	scFv-CD3 TM+IC	Glioblastoma	**/**	Alkins et al. (121)
HER2	NK92 cell line	scFv-CD28 TM+IC-CD3ζ	Glioblastoma	**/**	Zhang et al. (122)
HER2	NK92 cell line	scFv-CD28-CD3ζ	Glioblastoma	I	NCT03383978
HER2	NK92 cell line	Unknown	Glioblastoma	I	NCT03383978
MUC1	Unknown	Unknown	High Grade Glioma	I/II	NCT02839954
None	PBMCs	None	High Grade Glioma	I	NCT04254419
None	PBMCs	None	Glioma	I	NCT00909558
None	Placenta	None	High Grade Glioma	I	NCT04489420

## Future Perspectives

Compared to T cell-based therapy, the development of NK cell-based therapy falls behind to some degree in the treatment of glioma. Adoptive NK cell therapy is lack of *in vivo* persistence without cytokine support, which may limit the efficacy of the NK cell immunotherapy. System administration of cytokines is associated with undesirable toxicities as described above. Trafficking to tumor beds is critical for the efficacy of adoptive cellular therapy. Müler et al. observed an infiltration increase of anti-EGFRvIII CAR-NK cells engineered to express CXCR4 to CXCL12/SDF-1α secreting glioblastoma cells, leading to improved tumor regression and survival in a mouse model of glioblastoma (90). The difficulty may be resolved by intratumoral administration of NK cells products. In general, there is much room for the development of CAR-NK therapy in the field of glioma treatment. Much more pressing for NK cell-based therapy is designing more efficient products in treating glioma. Therefore, we try to put up several strategies to achieve the goal on the base of the knowledge of NK cells and glioma.

NK cell therapy is the lack of *in vivo* persistence in the absence of cytokine support. IL-15 is essential for NK cell function and homeostasis and can be added to CAR molecules to mimic the fourth generation of T-CAR. TGF-β plays an essential role in impairing NK cell function. Inverted CAR may be applied to reverse the situation by fusing the ectodomain of the TGF-β receptor to the endodomain of an activating receptor. CD3ζ is found in the intracellular domains of T-CAR and NK-CAR. Furthermore, CD3ζ, containing three immunoreceptor tyrosine-based activation motifs (ITAMs; YxxL/Ix6-8YxxL/I, with 29 amino acids), has a limited impact on the effectiveness of CAR-NK (123).

Modification targeting CD3ζ has been tested in CAR-T therapy. Wu et al. found that CD3ϵ recruits Csk and p85 *via* its mono-phosphorylated ITAM and BRS motif, respectively (124). Incorporation of the ITAM of CD3ϵ into a second-generation CAR increased the antitumor activity of CAR-T cells by reducing the cytokine production and promoting the persistence of CAR-T (124). NK cells possess many types of stimulatory receptors, such as CD16, NKp46, and NKG2D, and these stimulatory receptors do not act separately. In fact, apart from CD16, which is sufficient for activation of resting NK cells, it is necessary for all activating receptors to cooperate and synergize with one another for NK activation (125). Interestingly, the ITAM of many stimulatory receptors or their related adaptors, such as FcϵRIγ and DAP12, also consists of 29 amino acids with different sequences. Therefore, an exchange may produce more effective CAR-NK cells (**Figure 2E**) with mild changes in CD3ζ structure.

CAR-NK cells can kill targets in a CAR-independent manner. Combination therapy with monoclonal antibodies is promising, and it was observed that trifunctional antibodies recognized targets and simultaneously engaged NKP46 and CD16, which controlled tumor growth in mouse models (126). A team used antibodies to prevent the loss of cell surface MICA and MICB in human cancer cells, which stabilizes the bond between NKG2D and its ligands. These antibodies inhibit tumor growth in mouse models, and the antitumor effect is mediated mainly by the activation of NKG2D and CD16 (127). Apart from the activating receptors, antibodies blocking the inhibitory receptors of NK cells, such as KIR, NKG2A, TIM3, and TIGIT have been studied (52, 128–130). NK cells can express FcγRIIIA/CD16a and/or FcγRIIC, which bind to the Fc portion of human immunoglobulins. Once antibodies bind to targets, NK cells are able to recognize the Fc portion and lyse target cells through antibody-dependent cell-mediated cytotoxicity (ADCC) (131). So, the combination of immune checkpoint inhibitors and NK cell-based therapy may be potential. Although, Nivolumab failed to improve overall survival of patients with recurrent glioblastoma. Studies have found that cancer type 1 or 2 susceptibility gene (BRCA1/2) alteration was associated with higher tumor mutation burden(TMB) and may serve as a novel indicator associated with better treatment outcomes of immune checkpoint inhibitors (132). The function of BRCA1/2 and other DNA mismatch repair gene alteration are worth being investigated in glioma. Most GBM cells express high levels of MHC class I molecules (112). Thus, blockade of such KIRs with antibodies may enhance NK-cell mediated killing.

NK cells and T cells originate from a common ancestor and share many similarities. Both interact with MHC class I molecules, contributing to innate and adaptive immunity. They have similar cell-surface phenotypes and cellular functions, such as cytotoxicity, secretion of cytokines, and interaction with DCs (133). NK cells also play a role in regulating T cell response. For example, NK cells can produce IFN-gamma, which promotes CD4+ T cell differentiation into TH1 helper cells (134). The latter contributes to an enhanced CD8+ T cell response (135). NK cells produce IFN-gamma, leading to DC maturation and IL-12 secretion, which is sufficient for CD8+ T cell activation independent of CD4+ T cell (136). As an important constituent of the innate immunity response, NK cells can kill target cells and release antigen for cross-presentation and activation of T cells (137). NK cells can also negatively regulate a T cell response as described above or in the setting of acute viral infections (138, 139). A combination of NK cells and T cells comprise an ideal potential therapy for tumor treatment. A study has reported that CAR-NK cells can eliminate myeloid-derived suppressor cells and rescue impaired CAR-T cell activity against solid tumors (140). Moreover, CAR-NK cells can improve the infiltration and functions of subsequently infused CAR-T cells by secreting proinflammatory cytokines and chemokines (140). Cózar et al. found marked NK-cell infiltration in solid tumors were also infiltrated with T cells (117).Anti-IL13Rα, anti-HER2, and anti-EGFRvIII CAR-T have been tested in glioma (141–143). Utilizing the safety of CAR-NK cells and the high efficacy of CAR-T cells is worth exploring in gliomas.

Oncolytic virus (OV) OVs have a double oncolytic action by both directly attacking the cancer cells and inspiring a tumor specific immune response. OVs can be engineered to repress antibodies targeting tumor antigen and/or secret cytokines activating immune response. Xilin Chen et al. observed that the combination of EGFR-CAR NK-92 cells with oHSV-1 resulted in more efficient killing of MDA-MB-231 tumor cells and significantly longer survival of tumor-bearing mice (144). Rui Ma et al. (145) generated a therapy that combined off-the-shelf EGFR-CAR NK cells and an Oncolytic virus OV called OV-IL15C. OV-IL15C-infected GBM cells can secrete soluble IL15/IL15Rα complex. GBM-bearing mice models exhibited that the therapy synergistically suppressed tumor growth. These potential therapies are anticipated to be further investigated in clinical trials. Combination therapies are based on the knowledge of NK cell biology. An evolving understanding of gliomas can inspire treatment strategies targeting the basic elements of these malignant cells and their microenvironments. We believe NK cell-based immunotherapy will have a better performance in treating glioma in the future.

## Author Contributions

CP conceived the article. YZ compiled the review and prepared the draft of the manuscript. GL, TJ, and WZ reviewed and edited the manuscript. All authors contributed to the article and approved the submitted version.

## Funding

This work was supported by grants from National Natural Science Foundation of China (No.82072768), Construction Project of Multi Omics Platform for Major Brain Diseases (PXM2019_026280_000002), Sino German Cooperation and Exchange Project (Mobility Programme, M-0020), Research Fund for Clinical and Translational Medicine of Chinese Academy of Medical Sciences (2020-I2M-C&T-A-024).

## Conflict of Interest

The authors declare that the research was conducted in the absence of any commercial or financial relationships that could be construed as a potential conflict of interest.

## Publisher’s Note

All claims expressed in this article are solely those of the authors and do not necessarily represent those of their affiliated organizations, or those of the publisher, the editors and the reviewers. Any product that may be evaluated in this article, or claim that may be made by its manufacturer, is not guaranteed or endorsed by the publisher.

## References

[1] Janjua TI Rewatkar P Ahmed-Cox A Saeed I Mansfeld FM Kulshreshtha R. Frontiers in the Treatment of Glioblastoma: Past, Present and Emerging. Adv Drug Delivery Rev (2021) 171:108–38. doi: 10.1016/j.addr.2021.01.012 33486006

[2] Hamisch CA Minartz J Blau T Hafkemeyer V Rueß D Hellerbach A. Frame-Based Stereotactic Biopsy of Deep-Seated and Midline Structures in 511 Procedures: Feasibility, Risk Profile, and Diagnostic Yield. Acta Neurochir (Wien) (2019) 161(10):2065–71. doi: 10.1007/s00701-019-04020-1 31359191

[3] Buckner JC Shaw EG Pugh SL Chakravarti A Gilbert MR Barger GR. Radiation Plus Procarbazine, CCNU, and Vincristine in Low-Grade Glioma. N Engl J Med (2016) 374(14):1344–55. doi: 10.1056/NEJMoa1500925 PMC5170873 27050206

[4] Cloughesy TF Mochizuki AY Orpilla JR Hugo W Lee AH Davidson TB. Neoadjuvant Anti-PD-1 Immunotherapy Promotes a Survival Benefit With Intratumoral and Systemic Immune Responses in Recurrent Glioblastoma. Nat Med (2019) 25(3):477–86. doi: 10.1038/s41591-018-0337-7 PMC6408961 30742122

[5] June CH O’Connor RS Kawalekar OU Ghassemi S Milone MC. CAR T Cell Immunotherapy for Human Cancer. Science (2018) 359(6382):1361–5. doi: 10.1126/science.aar6711 29567707

[6] Kochenderfer JN Dudley ME Kassim SH Somerville RP Carpenter RO Stetler-Stevenson M. Chemotherapy-Refractory Diffuse Large B-Cell Lymphoma and Indolent B-Cell Malignancies can be Effectively Treated With Autologous T Cells Expressing an Anti-CD19 Chimeric Antigen Receptor. J Clin Oncol (2015) 33(6):540–9. doi: 10.1200/jco.2014.56.2025 PMC4322257 25154820

[7] Park JH Rivière I Gonen M Wang X Sénéchal B Curran KJ. Long-Term Follow-Up of CD19 CAR Therapy in Acute Lymphoblastic Leukemia. N Engl J Med (2018) 378(5):449–59. doi: 10.1056/NEJMoa1709919 PMC6637939 29385376

[8] Singh AK McGuirk JP. CAR T Cells: Continuation in a Revolution of Immunotherapy. Lancet Oncol (2020) 21(3):e168–e78. doi: 10.1016/s1470-2045(19)30823-x 32135120

[9] Vivier E Tomasello E Baratin M Walzer T Ugolini S. Functions of Natural Killer Cells. Nat Immunol (2008) 9(5):503–10. doi: 10.1038/ni1582 18425107

[10] Zhang Y Wallace DL de Lara CM Ghattas H Asquith B Worth A. *In Vivo* Kinetics of Human Natural Killer Cells: The Effects of Ageing and Acute and Chronic Viral Infection. Immunology (2007) 121(2):258–65. doi: 10.1111/j.1365-2567.2007.02573.x PMC2265941 17346281

[11] Keating SE Zaiatz-Bittencourt V Loftus RM Keane C Brennan K Finlay DK. Metabolic Reprogramming Supports IFN-γ Production by CD56bright NK Cells. J Immunol (2016) 196(6):2552–60. doi: 10.4049/jimmunol.1501783 26873994

[12] Stringaris K Sekine T Khoder A Alsuliman A Razzaghi B Sargeant R. Leukemia-Induced Phenotypic and Functional Defects in Natural Killer Cells Predict Failure to Achieve Remission in Acute Myeloid Leukemia. Haematologica (2014) 99(5):836–47. doi: 10.3324/haematol.2013.087536 PMC4008119 24488563

[13] Rubnitz JE Inaba H Ribeiro RC Pounds S Rooney B Bell T. NKAML: A Pilot Study to Determine the Safety and Feasibility of Haploidentical Natural Killer Cell Transplantation in Childhood Acute Myeloid Leukemia. J Clin Oncol (2010) 28(6):955–9. doi: 10.1200/jco.2009.24.4590 PMC2834435 20085940

[14] Miller JS Soignier Y Panoskaltsis-Mortari A McNearney SA Yun GH Fautsch SK. Successful Adoptive Transfer and In Vivo Expansion of Human Haploidentical NK Cells in Patients With Cancer. Blood (2005) 105(8):3051–7. doi: 10.1182/blood-2004-07-2974 15632206

[15] Morvan MG Lanier LL. NK Cells and Cancer: You can Teach Innate Cells New Tricks. Nat Rev Cancer (2016) 16(1):7–19. doi: 10.1038/nrc.2015.5 26694935

[16] Liu E Marin D Banerjee P Macapinlac HA Thompson P Basar R. Use of CAR-Transduced Natural Killer Cells in CD19-Positive Lymphoid Tumors. N Engl J Med (2020) 382(6):545–53. doi: 10.1056/NEJMoa1910607 PMC7101242 32023374

[17] Colonna M Samaridis J. Cloning of Immunoglobulin-Superfamily Members Associated With HLA-C and HLA-B Recognition by Human Natural Killer Cells. Science (1995) 268(5209):405–8. doi: 10.1126/science.7716543 7716543

[18] Ruggeri L Mancusi A Capanni M Urbani E Carotti A Aloisi T. Donor Natural Killer Cell Allorecognition of Missing Self in Haploidentical Hematopoietic Transplantation for Acute Myeloid Leukemia: Challenging Its Predictive Value. Blood (2007) 110(1):433–40. doi: 10.1182/blood-2006-07-038687 PMC1896125 17371948

[19] Storkus WJ Howell DN Salter RD Dawson JR Cresswell P. NK Susceptibility Varies Inversely With Target Cell Class I HLA Antigen Expression. J Immunol (1987) 138(6):1657–9. 3819393

[20] Moretta A Vitale M Bottino C Orengo AM Morelli L Augugliaro R. P58 Molecules as Putative Receptors for Major Histocompatibility Complex (MHC) Class I Molecules in Human Natural Killer (NK) Cells. Anti-P58 Antibodies Reconstitute Lysis of MHC Class I-Protected Cells in NK Clones Displaying Different Specificities. J Exp Med (1993) 178(2):597–604. doi: 10.1084/jem.178.2.597 8340759 PMC2191136

[21] Pende D Falco M Vitale M Cantoni C Vitale C Munari E. Killer Ig-Like Receptors (KIRs): Their Role in NK Cell Modulation and Developments Leading to Their Clinical Exploitation. Front Immunol (2019) 101179:1179. doi: 10.3389/fimmu.2019.01179 PMC6558367 31231370

[22] Uhrberg M Valiante NM Shum BP Shilling HG Lienert-Weidenbach K Corliss B. Human Diversity in Killer Cell Inhibitory Receptor Genes. Immunity (1997) 7(6):753–63. doi: 10.1016/s1074-7613(00)80394-5 9430221

[23] Colonna M Brooks EG Falco M Ferrara GB Strominger JL. Generation of Allospecific Natural Killer Cells by Stimulation Across a Polymorphism of HLA-C. Science (1993) 260(5111):1121–4. doi: 10.1126/science.8493555 8493555

[24] López-Botet M Pérez-Villar JJ Carretero M Rodríguez A Melero I Bellón T. Structure and Function of the CD94 C-Type Lectin Receptor Complex Involved in Recognition of HLA Class I Molecules. Immunol Rev (1997) 155:165–74. doi: 10.1111/j.1600-065x.1997.tb00949.x 9059892

[25] Brooks AG Posch PE Scorzelli CJ Borrego F Coligan JE. NKG2A Complexed With CD94 Defines a Novel Inhibitory Natural Killer Cell Receptor. J Exp Med (1997) 185(4):795–800. doi: 10.1084/jem.185.4.795 9034158 PMC2196137

[26] Carretero M Cantoni C Bellón T Bottino C Biassoni R Rodríguez A. The CD94 and NKG2-A C-Type Lectins Covalently Assemble to Form a Natural Killer Cell Inhibitory Receptor for HLA Class I Molecules. Eur J Immunol (1997) 27(2):563–7. doi: 10.1002/eji.1830270230 9045931

[27] Kärre K Ljunggren HG Piontek G Kiessling R. Selective Rejection of H-2-Deficient Lymphoma Variants Suggests Alternative Immune Defence Strategy. Nature (1986) 319(6055):675–8. doi: 10.1038/319675a0 3951539

[28] Farag SS Fehniger TA Ruggeri L Velardi A Caligiuri MA. Natural Killer Cell Receptors: New Biology and Insights Into the Graft-*Versus*-Leukemia Effect. Blood (2002) 100(6):1935–47. doi: 10.1182/blood-2002-02-0350 12200350

[29] Kärre K. NK Cells, MHC Class I Molecules and the Missing Self. Scand J Immunol (2002) 55(3):221–8. doi: 10.1046/j.1365-3083.2002.01053.x 11940227

[30] Ruggeri L Capanni M Casucci M Volpi I Tosti A Perruccio K. Role of Natural Killer Cell Alloreactivity in HLA-Mismatched Hematopoietic Stem Cell Transplantation. Blood (1999) 94(1):333–9. doi: 10.1182/blood.V94.1.333.413a31_333_339 10381530

[31] Yabe T McSherry C Bach FH Fisch P Schall RP Sondel PM. A Multigene Family on Human Chromosome 12 Encodes Natural Killer-Cell Lectins. Immunogenetics (1993) 37(6):455–60. doi: 10.1007/bf00222470 8436421

[32] Gumperz JE Valiante NM Parham P Lanier LL Tyan D. Heterogeneous Phenotypes of Expression of the NKB1 Natural Killer Cell Class I Receptor Among Individuals of Different Human Histocompatibility Leukocyte Antigens Types Appear Genetically Regulated, But Not Linked to Major Histocompatibililty Complex Haplotype. J Exp Med (1996) 183(4):1817–27. doi: 10.1084/jem.183.4.1817 PMC2192483 8666938

[33] Vilches C Parham P. KIR: Diverse, Rapidly Evolving Receptors of Innate and Adaptive Immunity. Annu Rev Immunol (2002) 20:217–51. doi: 10.1146/annurev.immunol.20.092501.134942 11861603

[34] Hsu KC Keever-Taylor CA Wilton A Pinto C Heller G Arkun K. Improved Outcome in HLA-Identical Sibling Hematopoietic Stem-Cell Transplantation for Acute Myelogenous Leukemia Predicted by KIR and HLA Genotypes. Blood (2005) 105(12):4878–84. doi: 10.1182/blood-2004-12-4825 PMC1894998 15731175

[35] Leung W Iyengar R Triplett B Turner V Behm FG Holladay MS. Comparison of Killer Ig-Like Receptor Genotyping and Phenotyping for Selection of Allogeneic Blood Stem Cell Donors. J Immunol (2005) 174(10):6540–5. doi: 10.4049/jimmunol.174.10.6540 15879158

[36] Leung W Iyengar R Turner V Lang P Bader P Conn P. Determinants of Antileukemia Effects of Allogeneic NK Cells. J Immunol (2004) 172(1):644–50. doi: 10.4049/jimmunol.172.1.644 14688377

[37] Hsu KC Gooley T Malkki M Pinto-Agnello C Dupont B Bignon JD. KIR Ligands and Prediction of Relapse After Unrelated Donor Hematopoietic Cell Transplantation for Hematologic Malignancy. Biol Blood Marrow Transplant (2006) 12(8):828–36. doi: 10.1016/j.bbmt.2006.04.008 16864053

[38] Verheyden S Schots R Duquet W Demanet C. A Defined Donor Activating Natural Killer Cell Receptor Genotype Protects Against Leukemic Relapse After Related HLA-Identical Hematopoietic Stem Cell Transplantation. Leukemia (2005) 19(8):1446–51. doi: 10.1038/sj.leu.2403839 15973456

[39] Sconocchia G Lau M Provenzano M Rezvani K Wongsena W Fujiwara H. The Antileukemia Effect of HLA-Matched NK and NK-T Cells in Chronic Myelogenous Leukemia Involves NKG2D-Target-Cell Interactions. Blood (2005) 106(10):3666–72. doi: 10.1182/blood-2005-02-0479 PMC1895055 16046526

[40] Verheyden S Demanet C. NK Cell Receptors and Their Ligands in Leukemia. Leukemia (2008) 22(2):249–57. doi: 10.1038/sj.leu.2405040 18046448

[41] Shimasaki N Jain A Campana D. NK Cells for Cancer Immunotherapy. Nat Rev Drug Discovery (2020) 19(3):200–18. doi: 10.1038/s41573-019-0052-1 31907401

[42] Weiden PL Flournoy N Thomas ED Prentice R Fefer A Buckner CD. Antileukemic Effect of Graft-*Versus*-Host Disease in Human Recipients of Allogeneic-Marrow Grafts. N Engl J Med (1979) 300(19):1068–73. doi: 10.1056/nejm197905103001902 34792

[43] Horowitz MM Gale RP Sondel PM Goldman JM Kersey J Kolb HJ. Graft-*Versus*-Leukemia Reactions After Bone Marrow Transplantation. Blood (1990) 75(3):555–62. doi: 10.1182/blood.V75.3.555.555 2297567

[44] Ferrara JL Levine JE Reddy P Holler E. Graft-*Versus*-Host Disease. Lancet (2009) 373(9674):1550–61. doi: 10.1016/s0140-6736(09)60237-3 PMC2735047 19282026

[45] Giebel S Locatelli F Lamparelli T Velardi A Davies S Frumento G. Survival Advantage With KIR Ligand Incompatibility in Hematopoietic Stem Cell Transplantation From Unrelated Donors. Blood (2003) 102(3):814–9. doi: 10.1182/blood-2003-01-0091 12689936

[46] Ruggeri L Capanni M Urbani E Perruccio K Shlomchik WD Tosti A. Effectiveness of Donor Natural Killer Cell Alloreactivity in Mismatched Hematopoietic Transplants. Science (2002) 295(5562):2097–100. doi: 10.1126/science.1068440 11896281

[47] Cerboni C Zingoni A Cippitelli M Piccoli M Frati L Santoni A. Antigen-Activated Human T Lymphocytes Express Cell-Surface NKG2D Ligands *via* an ATM/ATR-Dependent Mechanism and Become Susceptible to Autologous NK- Cell Lysis. Blood (2007) 110(2):606–15. doi: 10.1182/blood-2006-10-052720 17405908

[48] Miller JS Cooley S Parham P Farag SS Verneris MR McQueen KL. Missing KIR Ligands are Associated With Less Relapse and Increased Graft-*Versus*-Host Disease (GVHD) Following Unrelated Donor Allogeneic HCT. Blood (2007) 109(11):5058–61. doi: 10.1182/blood-2007-01-065383 PMC1885526 17317850

[49] Barnes DW Corp MJ Loutit JF Neal FE. Treatment of Murine Leukaemia With X Rays and Homologous Bone Marrow; Preliminary Communication. Br Med J (1956) 2(4993):626–7. doi: 10.1136/bmj.2.4993.626 PMC2035298 13356034

[50] Bachar-Lustig E Rachamim N Li HW Lan F Reisner Y. Megadose of T Cell-Depleted Bone Marrow Overcomes MHC Barriers in Sublethally Irradiated Mice. Nat Med (1995) 1(12):1268–73. doi: 10.1038/nm1295-1268 7489407

[51] Valiante NM Uhrberg M Shilling HG Lienert-Weidenbach K Arnett KL D’Andrea A. Functionally and Structurally Distinct NK Cell Receptor Repertoires in the Peripheral Blood of Two Human Donors. Immunity (1997) 7(6):739–51. doi: 10.1016/s1074-7613(00)80393-3 9430220

[52] André P Denis C Soulas C Bourbon-Caillet C Lopez J Arnoux T. Anti-NKG2A mAb Is a Checkpoint Inhibitor That Promotes Anti-Tumor Immunity by Unleashing Both T and NK Cells. Cell (2018) 175(7):1731–43.e13. doi: 10.1016/j.cell.2018.10.014 30503213 PMC6292840

[53] Braud V Jones EY McMichael A. The Human Major Histocompatibility Complex Class Ib Molecule HLA-E Binds Signal Sequence-Derived Peptides With Primary Anchor Residues at Positions 2 and 9. Eur J Immunol (1997) 27(5):1164–9. doi: 10.1002/eji.1830270517 9174606

[54] Braud VM Allan DS Wilson D McMichael AJ. TAP- and Tapasin-Dependent HLA-E Surface Expression Correlates With the Binding of an MHC Class I Leader Peptide. Curr Biol (1998) 8(1):1–10. doi: 10.1016/s0960-9822(98)70014-4 9427624

[55] Bauer S Groh V Wu J Steinle A Phillips JH Lanier LL. Activation of NK Cells and T Cells by NKG2D, a Receptor for Stress-Inducible MICA. Science (1999) 285(5428):727–9. doi: 10.1126/science.285.5428.727 10426993

[56] Cosman D Müllberg J Sutherland CL Chin W Armitage R Fanslow W. ULBPs, Novel MHC Class I-Related Molecules, Bind to CMV Glycoprotein UL16 and Stimulate NK Cytotoxicity Through the NKG2D Receptor. Immunity (2001) 14(2):123–33. doi: 10.1016/s1074-7613(01)00095-4 11239445

[57] Fajgenbaum DC June CH. Cytokine Storm. N Engl J Med (2020) 383(23):2255–73. doi: 10.1056/NEJMra2026131 PMC7727315 33264547

[58] Klingemann H. Are Natural Killer Cells Superior CAR Drivers? Oncoimmunology (2014) 3:e28147. doi: 10.4161/onci.28147 25340009 PMC4203506

[59] Tang X Yang L Li Z Nalin AP Dai H Xu T. First-In-Man Clinical Trial of CAR NK-92 Cells: Safety Test of CD33-CAR NK-92 Cells in Patients With Relapsed and Refractory Acute Myeloid Leukemia. Am J Cancer Res (2018) 8(6):1083–9. PMC6048396 30034945

[60] Parkhurst MR Riley JP Dudley ME Rosenberg SA. Adoptive Transfer of Autologous Natural Killer Cells Leads to High Levels of Circulating Natural Killer Cells But Does Not Mediate Tumor Regression. Clin Cancer Res (2011) 17(19):6287–97. doi: 10.1158/1078-0432.Ccr-11-1347 PMC3186830 21844012

[61] Maki G Klingemann HG Martinson JA Tam YK. Factors Regulating the Cytotoxic Activity of the Human Natural Killer Cell Line, NK-92. J Hematother Stem Cell Res (2001) 10(3):369–83. doi: 10.1089/152581601750288975 11454312

[62] Schönfeld K Sahm C Zhang C Naundorf S Brendel C Odendahl M. Selective Inhibition of Tumor Growth by Clonal NK Cells Expressing an ErbB2/HER2-Specific Chimeric Antigen Receptor. Mol Ther (2015) 23(2):330–8. doi: 10.1038/mt.2014.219 PMC4445620 25373520

[63] Vela M Corral D Carrasco P Fernández L Valentín J González B. Haploidentical IL-15/41BBL Activated and Expanded Natural Killer Cell Infusion Therapy After Salvage Chemotherapy in Children With Relapsed and Refractory Leukemia. Cancer Lett (2018) 422:107–17. doi: 10.1016/j.canlet.2018.02.033 29477379

[64] Ciurea SO Schafer JR Bassett R Denman CJ Cao K Willis D. Phase 1 Clinical Trial Using Mbil21 Ex Vivo-Expanded Donor-Derived NK Cells After Haploidentical Transplantation. Blood (2017) 130(16):1857–68. doi: 10.1182/blood-2017-05-785659 PMC5649552 28835441

[65] Huntington ND Puthalakath H Gunn P Naik E Michalak EM Smyth MJ. Interleukin 15-Mediated Survival of Natural Killer Cells Is Determined by Interactions Among Bim, Noxa and Mcl-1. Nat Immunol (2007) 8(8):856–63. doi: 10.1038/ni1487 PMC2951739 17618288

[66] Guo Y Luan L Patil NK Sherwood ER. Immunobiology of the IL-15/IL-15rα Complex as an Antitumor and Antiviral Agent. Cytokine Growth Factor Rev (2017) 38:10–21. doi: 10.1016/j.cytogfr.2017.08.002 28888485 PMC5705392

[67] Robertson MJ Cameron C Lazo S Cochran KJ Voss SD Ritz J. Costimulation of Human Natural Killer Cell Proliferation: Role of Accessory Cytokines and Cell Contact-Dependent Signals. Nat Immun (1996) 15(5):213–26. 9390270

[68] Sakamoto N Ishikawa T Kokura S Okayama T Oka K Ideno M. Phase I Clinical Trial of Autologous NK Cell Therapy Using Novel Expansion Method in Patients With Advanced Digestive Cancer. J Transl Med (2015) 13:277. doi: 10.1186/s12967-015-0632-8 26303618 PMC4548900

[69] Ishikawa T Okayama T Sakamoto N Ideno M Oka K Enoki T. Phase I Clinical Trial of Adoptive Transfer of Expanded Natural Killer Cells in Combination With IgG1 Antibody in Patients With Gastric or Colorectal Cancer. Int J Cancer (2018) 142(12):2599–609. doi: 10.1002/ijc.31285 29388200

[70] Ruoslahti E. Fibronectin and its Receptors. Annu Rev Biochem (1988) 57:375–413. doi: 10.1146/annurev.bi.57.070188.002111 2972252

[71] Rabinovich BA Li J Shannon J Hurren R Chalupny J Cosman D. Activated, But Not Resting, T Cells can be Recognized and Killed by Syngeneic NK Cells. J Immunol (2003) 170(7):3572–6. doi: 10.4049/jimmunol.170.7.3572 12646619

[72] Lee HR Son CH Koh EK Bae JH Kang CD Yang K. Expansion of Cytotoxic Natural Killer Cells Using Irradiated Autologous Peripheral Blood Mononuclear Cells and Anti-CD16 Antibody. Sci Rep (2017) 7(1):11075. doi: 10.1038/s41598-017-09259-1 28894091 PMC5593981

[73] Miller JS Oelkers S Verfaillie C McGlave P. Role of Monocytes in the Expansion of Human Activated Natural Killer Cells. Blood (1992) 80(9):2221–9. doi: 10.1182/blood.V80.9.2221.2221 1421393

[74] Li L Li W Wang C Yan X Wang Y Niu C. Adoptive Transfer of Natural Killer Cells in Combination With Chemotherapy Improves Outcomes of Patients With Locally Advanced Colon Carcinoma. Cytotherapy (2018) 20(1):134–48. doi: 10.1016/j.jcyt.2017.09.009 29056549

[75] Lehrnbecher T Foster CB Zhu S Leitman SF Goldin LR Huppi K. Variant Genotypes of the Low-Affinity Fcgamma Receptors in Two Control Populations and a Review of Low-Affinity Fcgamma Receptor Polymorphisms in Control and Disease Populations. Blood (1999) 94(12):4220–32. doi: 10.1182/blood.V94.12.4220.424k08_4220_4232 10590067

[76] Freud AG Mundy-Bosse BL Yu J Caligiuri MA. The Broad Spectrum of Human Natural Killer Cell Diversity. Immunity (2017) 47(5):820–33. doi: 10.1016/j.immuni.2017.10.008 PMC5728700 29166586

[77] Grimm EA Mazumder A Zhang HZ Rosenberg SA. Lymphokine-Activated Killer Cell Phenomenon. Lysis of Natural Killer-Resistant Fresh Solid Tumor Cells by Interleukin 2-Activated Autologous Human Peripheral Blood Lymphocytes. J Exp Med (1982) 155(6):1823–41. doi: 10.1084/jem.155.6.1823 PMC2186695 6176669

[78] Rosenberg SA Spiess P Lafreniere R. A New Approach to the Adoptive Immunotherapy of Cancer With Tumor-Infiltrating Lymphocytes. Science (1986) 233(4770):1318–21. doi: 10.1126/science.3489291 3489291

[79] Restifo NP Dudley ME Rosenberg SA. Adoptive Immunotherapy for Cancer: Harnessing the T Cell Response. Nat Rev Immunol (2012) 12(4):269–81. doi: 10.1038/nri3191 PMC6292222 22437939

[80] Zacharakis N Chinnasamy H Black M Xu H Lu YC Zheng Z. Immune Recognition of Somatic Mutations Leading to Complete Durable Regression in Metastatic Breast Cancer. Nat Med (2018) 24(6):724–30. doi: 10.1038/s41591-018-0040-8 PMC6348479 29867227

[81] Morgan RA Dudley ME Wunderlich JR Hughes MS Yang JC Sherry RM. Cancer Regression in Patients After Transfer of Genetically Engineered Lymphocytes. Science (2006) 314(5796):126–9. doi: 10.1126/science.1129003 PMC2267026 16946036

[82] Zhai Y Li G Jiang T Zhang W. CAR-Armed Cell Therapy for Gliomas. Am J Cancer Res (2019) 9(12):2554–66. PMC6943349 31911846

[83] Leen AM Sukumaran S Watanabe N Mohammed S Keirnan J Yanagisawa R. Reversal of Tumor Immune Inhibition Using a Chimeric Cytokine Receptor. Mol Ther (2014) 22(6):1211–20. doi: 10.1038/mt.2014.47 PMC4048899 24732709

[84] Wang Y Jiang H Luo H Sun Y Shi B Sun R. An IL-4/21 Inverted Cytokine Receptor Improving CAR-T Cell Potency in Immunosuppressive Solid-Tumor Microenvironment. Front Immunol (2019) 10:1691. doi: 10.3389/fimmu.2019.01691 31379876 PMC6658891

[85] Han X Wang Y Wei J Han W. Multi-Antigen-Targeted Chimeric Antigen Receptor T Cells for Cancer Therapy. J Hematol Oncol (2019) 12(1):128. doi: 10.1186/s13045-019-0813-7 31783889 PMC6884912

[86] Xie G Dong H Liang Y Ham JD Rizwan R Chen J. CAR-NK Cells: A Promising Cellular Immunotherapy for Cancer. EBioMedicine (2020) 59:102975. doi: 10.1016/j.ebiom.2020.102975 32853984 PMC7452675

[87] Klöß S Oberschmidt O Morgan M Dahlke J Arseniev L Huppert V. Optimization of Human NK Cell Manufacturing: Fully Automated Separation, Improved Ex Vivo Expansion Using IL-21 With Autologous Feeder Cells, and Generation of Anti-CD123-CAR-Expressing Effector Cells. Hum Gene Ther (2017) 28(10):897–913. doi: 10.1089/hum.2017.157 28810809

[88] Tseng HC Xiong W Badeti S Yang Y Ma M Liu T. Efficacy of Anti-CD147 Chimeric Antigen Receptors Targeting Hepatocellular Carcinoma. Nat Commun (2020) 11(1):4810. doi: 10.1038/s41467-020-18444-2 32968061 PMC7511348

[89] Altvater B Landmeier S Pscherer S Temme J Schweer K Kailayangiri S. 2b4 (CD244) Signaling by Recombinant Antigen-Specific Chimeric Receptors Costimulates Natural Killer Cell Activation to Leukemia and Neuroblastoma Cells. Clin Cancer Res (2009) 15(15):4857–66. doi: 10.1158/1078-0432.Ccr-08-2810 PMC2771629 19638467

[90] Müller N Michen S Tietze S Töpfer K Schulte A Lamszus K. Engineering NK Cells Modified With an EGFRvIII-Specific Chimeric Antigen Receptor to Overexpress CXCR4 Improves Immunotherapy of CXCL12/SDF-1α-Secreting Glioblastoma. J Immunother (2015) 38(5):197–210. doi: 10.1097/cji.0000000000000082 25962108 PMC4428685

[91] Li Y Hermanson DL Moriarity BS Kaufman DS. Human iPSC-Derived Natural Killer Cells Engineered With Chimeric Antigen Receptors Enhance Anti-Tumor Activity. Cell Stem Cell (2018) 23(2):181–92.e5. doi: 10.1016/j.stem.2018.06.002 30082067 PMC6084450

[92] Billadeau DD Upshaw JL Schoon RA Dick CJ Leibson PJ. NKG2D-DAP10 Triggers Human NK Cell-Mediated Killing via a Syk-Independent Regulatory Pathway. Nat Immunol (2003) 4(6):557–64. doi: 10.1038/ni929 12740575

[93] Bryceson YT March ME Ljunggren HG Long EO. Activation, Coactivation, and Costimulation of Resting Human Natural Killer Cells. Immunol Rev (2006) 214:73–91. doi: 10.1111/j.1600-065X.2006.00457.x 17100877 PMC3845883

[94] Eissmann P Beauchamp L Wooters J Tilton JC Long EO Watzl C. Molecular Basis for Positive and Negative Signaling by the Natural Killer Cell Receptor 2B4 (CD244). Blood (2005) 105(12):4722–9. doi: 10.1182/blood-2004-09-3796 15713798

[95] Chakravarthy A Furness A Joshi K Ghorani E Ford K Ward MJ. Pan-Cancer Deconvolution of Tumour Composition Using DNA Methylation. Nat Commun (2018) 9(1):3220. doi: 10.1038/s41467-018-05570-1 30104673 PMC6089972

[96] Mahaley MS Jr. Gentry RE Bigner DD. Immunobiology of Primary Intracranial Tumors. J Neurosurg (1977) 47(1):35–43. doi: 10.3171/jns.1977.47.1.0035 194022

[97] Elliott LH Brooks WH Roszman TL. Activation of Immunoregulatory Lymphocytes Obtained From Patients With Malignant Gliomas. J Neurosurg (1987) 67(2):231–6. doi: 10.3171/jns.1987.67.2.0231 2885402

[98] McVicar DW Davis DF Merchant RE. *In Vitro* Analysis of the Proliferative Potential of T Cells From Patients With Brain Tumor: Glioma-Associated Immunosuppression Unrelated to Intrinsic Cellular Defect. J Neurosurg (1992) 76(2):251–60. doi: 10.3171/jns.1992.76.2.0251 1730954

[99] Ashkenazi E Deutsch M Tirosh R Weinreb A Tsukerman A Brodie C. A Selective Impairment of the IL-2 System in Lymphocytes of Patients With Glioblastomas: Increased Level of Soluble IL-2R and Reduced Protein Tyrosine Phosphorylation. Neuroimmunomodulation (1997) 4(1):49–56. doi: 10.1159/000097315 9326745

[100] Morford LA Elliott LH Carlson SL Brooks WH Roszman TL. T Cell Receptor-Mediated Signaling is Defective in T Cells Obtained From Patients With Primary Intracranial Tumors. J Immunol (1997) 159(9):4415–25. 9379040

[101] Raychaudhuri B Rayman P Ireland J Ko J Rini B Borden EC. Myeloid-Derived Suppressor Cell Accumulation and Function in Patients With Newly Diagnosed Glioblastoma. Neuro Oncol (2011) 13(6):591–9. doi: 10.1093/neuonc/nor042 PMC3107102 21636707

[102] Rodrigues JC Gonzalez GC Zhang L Ibrahim G Kelly JJ Gustafson MP. Normal Human Monocytes Exposed to Glioma Cells Acquire Myeloid-Derived Suppressor Cell-Like Properties. Neuro Oncol (2010) 12(4):351–65. doi: 10.1093/neuonc/nop023 PMC2940603 20308313

[103] Sinha P Clements VK Bunt SK Albelda SM Ostrand-Rosenberg S. Cross-Talk Between Myeloid-Derived Suppressor Cells and Macrophages Subverts Tumor Immunity Toward a Type 2 Response. J Immunol (2007) 179(2):977–83. doi: 10.4049/jimmunol.179.2.977 17617589

[104] Friese MA Wischhusen J Wick W Weiler M Eisele G Steinle A. RNA Interference Targeting Transforming Growth Factor-Beta Enhances NKG2D-Mediated Antiglioma Immune Response, Inhibits Glioma Cell Migration and Invasiveness, and Abrogates Tumorigenicity *In Vivo* . Cancer Res (2004) 64(20):7596–603. doi: 10.1158/0008-5472.Can-04-1627 15492287

[105] Castriconi R Cantoni C Della Chiesa M Vitale M Marcenaro E Conte R. Transforming Growth Factor Beta 1 Inhibits Expression of NKp30 and NKG2D Receptors: Consequences for the NK-Mediated Killing of Dendritic Cells. Proc Natl Acad Sci USA (2003) 100(7):4120–5. doi: 10.1073/pnas.0730640100 PMC153058 12646700

[106] Viel S Marçais A Guimaraes FS Loftus R Rabilloud J Grau M. TGF-β Inhibits the Activation and Functions of NK Cells by Repressing the mTOR Pathway. Sci Signal (2016) 9(415):ra19. doi: 10.1126/scisignal.aad1884 26884601

[107] Close HJ Stead LF Nsengimana J Reilly KA Droop A Wurdak H. Expression Profiling of Single Cells and Patient Cohorts Identifies Multiple Immunosuppressive Pathways and an Altered NK Cell Phenotype in Glioblastoma. Clin Exp Immunol (2020) 200(1):33–44. doi: 10.1111/cei.13403 31784984 PMC7066386

[108] Wei J Barr J Kong LY Wang Y Wu A Sharma AK. Glioblastoma Cancer-Initiating Cells Inhibit T-Cell Proliferation and Effector Responses by the Signal Transducers and Activators of Transcription 3 Pathway. Mol Cancer Ther (2010) 9(1):67–78. doi: 10.1158/1535-7163.Mct-09-0734 20053772 PMC2939737

[109] Kinjyo I Inoue H Hamano S Fukuyama S Yoshimura T Koga K. Loss of SOCS3 in T Helper Cells Resulted in Reduced Immune Responses and Hyperproduction of Interleukin 10 and Transforming Growth Factor-Beta 1. J Exp Med (2006) 203(4):1021–31. doi: 10.1084/jem.20052333 PMC2118269 16606674

[110] Laudati E Currò D Navarra P Lisi L. Blockade of CCR5 Receptor Prevents M2 Microglia Phenotype in a Microglia-Glioma Paradigm. Neurochem Int (2017) 108:100–8. doi: 10.1016/j.neuint.2017.03.002 28279751

[111] Held-Feindt J Hattermann K Müerköster SS Wedderkopp H Knerlich-Lukoschus F Ungefroren H. CX3CR1 Promotes Recruitment of Human Glioma-Infiltrating Microglia/Macrophages (GIMs). Exp Cell Res (2010) 316(9):1553–66. doi: 10.1016/j.yexcr.2010.02.018 20184883

[112] Golán I Rodríguez de la Fuente L Costoya JA. NK Cell-Based Glioblastoma Immunotherapy. Cancers (Basel) (2018) 10(12):522. doi: 10.3390/cancers10120522 PMC6315402 30567306

[113] O’Brien KL Finlay DK. Immunometabolism and Natural Killer Cell Responses. Nat Rev Immunol (2019) 19(5):282–90. doi: 10.1038/s41577-019-0139-2 30808985

[114] Tassinari V Cesarini V Tomaselli S Ianniello Z Silvestris DA Ginistrelli LC. ADAR1 is a New Target of METTL3 and Plays a Pro-Oncogenic Role in Glioblastoma by an Editing-Independent Mechanism. Genome Biol (2021) 22(1):51. doi: 10.1186/s13059-021-02271-9 33509238 PMC7842030

[115] Chang YZ Chai RC Pang B Chang X An SY Zhang KN. METTL3 Enhances the Stability of MALAT1 With the Assistance of HuR *via* M6a Modification and Activates NF-κb to Promote the Malignant Progression of IDH-Wildtype Glioma. Cancer Lett (2021) 511:36–46. doi: 10.1016/j.canlet.2021.04.020 33933553

[116] Zhou Z Zhang J Xu C Yang J Zhang Y Liu M. An Integrated Model of N6-Methyladenosine Regulators to Predict Tumor Aggressiveness and Immune Evasion in Pancreatic Cancer. EBioMedicine (2021) 65:103271. doi: 10.1016/j.ebiom.2021.103271 33714027 PMC7966986

[117] Cózar B Greppi M Carpentier S Narni-Mancinelli E Chiossone L Vivier E. Tumor-Infiltrating Natural Killer Cells. Cancer Discovery (2021) 11(1):34–44. doi: 10.1158/2159-8290.Cd-20-0655 33277307 PMC7611243

[118] Genßler S Burger MC Zhang C Oelsner S Mildenberger I Wagner M. Dual Targeting of Glioblastoma With Chimeric Antigen Receptor-Engineered Natural Killer Cells Overcomes Heterogeneity of Target Antigen Expression and Enhances Antitumor Activity and Survival. Oncoimmunology (2016) 5(4):e1119354. doi: 10.1080/2162402x.2015.1119354 27141401 PMC4839317

[119] Murakami T Nakazawa T Natsume A Nishimura F Nakamura M Matsuda R. Novel Human NK Cell Line Carrying CAR Targeting EGFRvIII Induces Antitumor Effects in Glioblastoma Cells. Anticancer Res (2018) 38(9):5049–56. doi: 10.21873/anticanres.12824 30194149

[120] Han J Chu J Keung Chan W Zhang J Wang Y Cohen JB. CAR-Engineered NK Cells Targeting Wild-Type EGFR and EGFRvIII Enhance Killing of Glioblastoma and Patient-Derived Glioblastoma Stem Cells. Sci Rep (2015) 5:11483. doi: 10.1038/srep11483 26155832 PMC4496728

[121] Alkins R Burgess A Kerbel R Wels WS Hynynen K. Early Treatment of HER2-Amplified Brain Tumors With Targeted NK-92 Cells and Focused Ultrasound Improves Survival. Neuro Oncol (2016) 18(7):974–81. doi: 10.1093/neuonc/nov318 PMC4896543 26819443

[122] Zhang C Burger MC Jennewein L Genßler S Schönfeld K Zeiner P. ErbB2/HER2-Specific NK Cells for Targeted Therapy of Glioblastoma. J Natl Cancer Inst (2016) 108(5). doi: 10.1093/jnci/djv375 26640245

[123] Burger MC Zhang C Harter PN Romanski A Strassheimer F Senft C. CAR-Engineered NK Cells for the Treatment of Glioblastoma: Turning Innate Effectors Into Precision Tools for Cancer Immunotherapy. Front Immunol (2019) 10:2683. doi: 10.3389/fimmu.2019.02683 31798595 PMC6868035

[124] Wu W Zhou Q Masubuchi T Shi X Li H Xu X. Multiple Signaling Roles of CD3ϵ and Its Application in CAR-T Cell Therapy. Cell (2020) 182(4):855–71.e23. doi: 10.1016/j.cell.2020.07.018 32730808

[125] Bryceson YT March ME Ljunggren HG Long EO. Synergy Among Receptors on Resting NK Cells for the Activation of Natural Cytotoxicity and Cytokine Secretion. Blood (2006) 107(1):159–66. doi: 10.1182/blood-2005-04-1351 PMC1895346 16150947

[126] Gauthier L Morel A Anceriz N Rossi B Blanchard-Alvarez A Grondin G. Multifunctional Natural Killer Cell Engagers Targeting NKp46 Trigger Protective Tumor Immunity. Cell (2019) 177(7):1701–13.e16. doi: 10.1016/j.cell.2019.04.041 31155232

[127] Ferrari de Andrade L Tay RE Pan D Luoma AM Ito Y Badrinath S. Antibody-Mediated Inhibition of MICA and MICB Shedding Promotes NK Cell-Driven Tumor Immunity. Science (2018) 359(6383):1537–42. doi: 10.1126/science.aao0505 PMC6626532 29599246

[128] da Silva IP Gallois A Jimenez-Baranda S Khan S Anderson AC Kuchroo VK. Reversal of NK-Cell Exhaustion in Advanced Melanoma by Tim-3 Blockade. Cancer Immunol Res (2014) 2(5):410–22. doi: 10.1158/2326-6066.Cir-13-0171 PMC4046278 24795354

[129] Zhang Q Bi J Zheng X Chen Y Wang H Wu W. Blockade of the Checkpoint Receptor TIGIT Prevents NK Cell Exhaustion and Elicits Potent Anti-Tumor Immunity. Nat Immunol (2018) 19(7):723–32. doi: 10.1038/s41590-018-0132-0 29915296

[130] Benson DM Jr Hofmeister CC Padmanabhan S Suvannasankha A Jagannath S Abonour R. A Phase 1 Trial of the Anti-KIR Antibody IPH2101 in Patients With Relapsed/Refractory Multiple Myeloma. Blood (2012) 120(22):4324–33. doi: 10.1182/blood-2012-06-438028 PMC3507143 23033266

[131] Wang W Erbe AK Hank JA Morris ZS Sondel PM. NK Cell-Mediated Antibody-Dependent Cellular Cytotoxicity in Cancer Immunotherapy. Front Immunol (2015) 6:368. doi: 10.3389/fimmu.2015.00368 26284063 PMC4515552

[132] Zhou Z Li M. Evaluation of BRCA1 and BRCA2 as Indicators of Response to Immune Checkpoint Inhibitors. JAMA Netw Open (2021) 4(5):e217728. doi: 10.1001/jamanetworkopen.2021.7728 33961040 PMC8105747

[133] Parham P. MHC Class I Molecules and KIRs in Human History, Health and Survival. Nat Rev Immunol (2005) 5(3):201–14. doi: 10.1038/nri1570 15719024

[134] Martín-Fontecha A Thomsen LL Brett S Gerard C Lipp M Lanzavecchia A. Induced Recruitment of NK Cells to Lymph Nodes Provides IFN-Gamma for T(H)1 Priming. Nat Immunol (2004) 5(12):1260–5. doi: 10.1038/ni1138 15531883

[135] Mocikat R Braumüller H Gumy A Egeter O Ziegler H Reusch U. Natural Killer Cells Activated by MHC Class I(low) Targets Prime Dendritic Cells to Induce Protective CD8 T Cell Responses. Immunity (2003) 19(4):561–9. doi: 10.1016/s1074-7613(03)00264-4 14563320

[136] Adam C King S Allgeier T Braumüller H Lüking C Mysliwietz J. DC-NK Cell Cross Talk as a Novel CD4+ T-Cell-Independent Pathway for Antitumor CTL Induction. Blood (2005) 106(1):338–44. doi: 10.1182/blood-2004-09-3775 15769894

[137] Krebs P Barnes MJ Lampe K Whitley K Bahjat KS Beutler B. NK-Cell-Mediated Killing of Target Cells Triggers Robust Antigen-Specific T-Cell-Mediated and Humoral Responses. Blood (2009) 113(26):6593–602. doi: 10.1182/blood-2009-01-201467 PMC2710917 19406986

[138] Cook KD Whitmire JK. The Depletion of NK Cells Prevents T Cell Exhaustion to Efficiently Control Disseminating Virus Infection. J Immunol (2013) 190(2):641–9. doi: 10.4049/jimmunol.1202448 PMC3879798 23241878

[139] Andrews DM Estcourt MJ Andoniou CE Wikstrom ME Khong A Voigt V. Innate Immunity Defines the Capacity of Antiviral T Cells to Limit Persistent Infection. J Exp Med (2010) 207(6):1333–43. doi: 10.1084/jem.20091193 PMC2882831 20513749

[140] Parihar R Rivas C Huynh M Omer B Lapteva N Metelitsa LS. NK Cells Expressing a Chimeric Activating Receptor Eliminate MDSCs and Rescue Impaired CAR-T Cell Activity Against Solid Tumors. Cancer Immunol Res (2019) 7(3):363–75. doi: 10.1158/2326-6066.Cir-18-0572 PMC7906796 30651290

[141] Brown CE Alizadeh D Starr R Weng L Wagner JR Naranjo A. Regression of Glioblastoma After Chimeric Antigen Receptor T-Cell Therapy. N Engl J Med (2016) 375(26):2561–9. doi: 10.1056/NEJMoa1610497 PMC5390684 28029927

[142] Ahmed N Brawley V Hegde M Bielamowicz K Kalra M Landi D. HER2-Specific Chimeric Antigen Receptor-Modified Virus-Specific T Cells for Progressive Glioblastoma: A Phase 1 Dose-Escalation Trial. JAMA Oncol (2017) 3(8):1094–101. doi: 10.1001/jamaoncol.2017.0184 PMC5747970 28426845

[143] O’Rourke DM Nasrallah MP Desai A Melenhorst JJ Mansfield K Morrissette JJD. A Single Dose of Peripherally Infused EGFRvIII-Directed CAR T Cells Mediates Antigen Loss and Induces Adaptive Resistance in Patients With Recurrent Glioblastoma. Sci Transl Med (2017) 9(399):eaaa0984. doi: 10.1126/scitranslmed.aaa0984 28724573 PMC5762203

[144] Chen X Han J Chu J Zhang L Zhang J Chen C. A Combinational Therapy of EGFR-CAR NK Cells and Oncolytic Herpes Simplex Virus 1 for Breast Cancer Brain Metastases. Oncotarget (2016) 7(19):27764–77. doi: 10.18632/oncotarget.8526 PMC5053686 27050072

[145] Ma R Lu T Li Z Teng KY Mansour AG Yu M. An Oncolytic Virus Expressing Il15/Il15rα Combined With Off-The-Shelf EGFR-CAR NK Cells Targets Glioblastoma. Cancer Res (2021) 81(13):3635–48. doi: 10.1158/0008-5472.Can-21-0035 PMC8562586 34006525

